# Accelerated epigenetic aging in suicide attempters uninfluenced by high intent-to-die and choice of lethal methods

**DOI:** 10.1038/s41398-022-01998-8

**Published:** 2022-06-02

**Authors:** Jussi Jokinen, Peter Andersson, Andreas Chatzittofis, Josephine Savard, Mathias Rask-Andersen, Marie Åsberg, Adrian Desai E. Boström

**Affiliations:** 1 Department of Clinical Sciences/Psychiatry, Umeå University, Umeå, Sweden; 2 Department of Clinical Neuroscience/Psychology, Karolinska Institute, Stockholm, Sweden; 3 Centre for Clinical Research Dalarna, Uppsala University, Falun, Sweden; 4 Medical School, University of Cyprus, Nicosia, Cyprus; 5 Department of Immunology, Genetics and Pathology, Science for Life Laboratory, Uppsala University, Uppsala, Sweden; 6 Department of Women’s and Children’s Health/Neuropediatrics, Karolinska Institutet, Stockholm, Sweden

**Keywords:** Psychiatric disorders, Epigenetics in the nervous system

## Abstract

Suicide attempts (SA) are associated with excess non-suicidal mortality, putatively mediated in part by premature cellular senescence. Epigenetic age (EA) estimators of biological age have been previously demonstrated to strongly predict physiological dysregulation and mortality risk. Herein, we investigate if violent SA with high intent-to-die is predictive of epigenetics-derived estimates of biological aging. The genome-wide methylation pattern was measured using the Illumina Infinium Methylation EPIC BeadChip in whole blood of 88 suicide attempters. Subjects were stratified into two groups based on the putative risk of later committed suicide (low- [*n* = 58] and high-risk [*n* = 30]) in dependency of SA method (violent or non-violent) and/or intent-to-die (high/low). Estimators of intrinsic and extrinsic EA acceleration, one marker optimized to predict physiological dysregulation (DNAmPhenoAge/AgeAccelPheno) and one optimized to predict lifespan (DNAmGrimAge/AgeAccelGrim) were investigated for associations to severity of SA, by univariate and multivariate analyses. The study was adequately powered to detect differences of 2.2 years in AgeAccelGrim in relation to SA severity. Baseline DNAmGrimAge exceeded chronological age by 7.3 years on average across all samples, conferring a mean 24.6% increase in relation to actual age. No individual EA acceleration marker was differentiated by suicidal risk group (*p* > 0.1). Thus, SA per se but not severity of SA is related to EA, implicating that excess non-suicidal mortality in SA is unrelated to risk of committed suicide. Preventative healthcare efforts aimed at curtailing excess mortality after SA may benefit from acting equally powerful to recognize somatic comorbidities irrespective of the severity inherent in the act itself.

## Introduction

Mental disorders confer substantial increases in all-cause and suicide mortality, greater than those associated with heavy smoking [1]. Effects may be partially mediated by disadvantageous lifestyle factors that impair physical health, e.g., substance or tobacco use, unfavorable dietary patterns or more sedentary behavior [2, 3]. These adverse health outcomes are common in suicide attempters, who exhibit increased life-time risks of contracting physical illnesses [4, 5]. Compared to the general population, young adults presenting with the first attempted suicide exhibit substantial reductions in life expectancies (between 11–20 years, depending on gender). Even though the suicide risk is high, the excess mortality is mainly explained by somatic comorbidities [6]. Emerging studies implicate premature cellular senescence to contribute to reductions in life-expectancies, which are observed in mental disorders [7]. Specifically, telomere length—a putative biomarker of accelerated aging and mortality—has been recently demonstrated to be shorter in suicide attempters with affective disorders [8]. Yet, suicide attempt is a highly heterogenous phenotype and prospective clinical and epidemiological studies demonstrate that high intent to die and a choice of a violent method, especially hanging, predict a higher risk of committed suicide [9, 10]. There is evidence for distinctive neurobiological underpinnings in severe suicidal behavior [11–13]. Consequently, research suggests that suicide victims are more comparable to high-intent or violent attempters as compared to non-violent attempters [14]. Thus, it can be argued that a lack of deep phenotyping and stratification of suicide attempters in these studies would prevent causal inferences. Even though severe suicide attempt predicts higher suicide risk, to our knowledge, no previous study investigated whether severe suicide attempt is associated with higher risk of natural-cause mortality. Since the causes of death are competing and most suicides occur within one year of the index attempt, one way to study this is to assess the epigenetic age in phenotypically well-characterized suicide attempters.

Advances in the study of DNA methylation—a key epigenetic process - has made possible the calculation of biological age, which, in comparison to chronological age, constitutes a concept more closely related to overall health status and cellular senescence [15]. Where the calculated biological age exceeds the chronological age, accelerated biological aging is deduced. A growing number of studies are making use of DNA methylation data to gauge the prospect of biological age acceleration in severe mental disorders by means of epigenetic age (EpiAge). However, causal inferences are so far prevented by insufficient power to produce substantive conclusions, ambiguity in interpreting different measures of EpiAge and inadequate deep phenotyping of subjects [16]. Indeed, a number of different “epigenetic clocks” has been presented (i.e. for example, Horvath’s Clock [17], Hannum Age [18], DNAm PhenoAge [19])—many successful in predicting chronological age, aspects of morbidity and excess mortality [17–20] yet not adequately correlated amongst themselves [20, 21]. This “epigenetic clock” conundrum, not yet fully elucidated, suggests the basis of biological aging is formed by several different processes, with each EpiAge estimator putatively reflecting a different aspect of biological aging [22]. By contrast, Lu et al. recently presented the “DNAm GrimAge” EpiAge clock (GrimAge). GrimAge is the result of consolidated surrogate DNA methylation levels used to estimate plasma protein levels and has been previously described as a powerful predictor of lifespan [20]. In a comparison with other EpiAge algorithms using three validation datasets comprising >7000 array measurements, GrimAge was demonstrably superior to all other algorithms studied in prediction of time-do-death, time-to-cancer (any) and time-to- coronary heart disease(CHD), while exhibiting strong relationship to radiology measured estimates of excess visceral fat and a comorbidity index (defined as the total number of age-related conditions) [20]. AgeAccelGrim—a measure of epigenetic age acceleration obtained by adjusting GrimAge to chronological age - is highly predictive of ageing-associated diseases including physical functioning, time-to-CHD, time-to-congestive heart failure, hypertension and type 2 diabetes mellitus [20].

Okazaki et al. recently demonstrated accelerated extrinsic epigenetic aging in suicide completers with proclaimed associations to the pathophysiology of suicide completion [23]. Importantly, the authors considered only intrinsic epigenetic age acceleration by Horvath (IEAA) [17] and extrinsic epigenetic age acceleration (EEAA) [17] as measures of EpiAge—both previously demonstrated not to correlate with age acceleration estimate developed by Lu et al. (AgeAccelGrim [20]) in psychiatric cohorts [22]—thus preventing any assured inferences regarding excess mortality. AgeAccelGrim has not yet been investigated in relation to severity of suicide attempt, even though suicide attempts are associated with excess somatic morbidity and mortality [4, 5]. In this study, we unconditionally investigate whether epigenetic aging using AgeAccelGrim is associated with severity of suicide attempt in a well-characterized cohort of suicide attempters – hypothesized as a plausible surrogate variable to assess whether severe suicide attempters exhibit excess morbidity and mortality conferred by cellular senescence.

## Methods

### Study population and ethics

Details on the suicide cohort and stratification of subjects have been previously published [11]. In brief, the study protocols were approved by the Regional Ethical Review Board in Stockholm, Sweden (Dnrs: 00-194, 2015/1454-32) and the participants gave their written informed consent to the study. The study sample includes adult patients that were clinically assessed at a Suicide Prevention Clinic (Karolinska University Hospital) in 2000–2005 after a suicide attempt with varying degrees of documented intention to die. Participant exclusion criteria included psychotic disorders, dementia, and mental retardation. As Stockholm Region at the time of recruitment provided specialized care for intravenous substance abusers at a tertiary non-affiliated clinic, these were not included in the study. However, suicide attempters often exhibit a present or past history of non-suicidal self-injury (NSSI) and/or substance abuse [24]. To improve the clinical generalizability of the study, NSSI and non-intravenous substance abuse (including alcohol dependency) did not constitute participant exclusion criteria. During the recruitment period, a total of 258 patients (89 male and 169 female patients) were assessed at the Suicide Prevention Clinic after a suicide attempt (including both in- and outpatients). Of these, 50 patients declined to participate, 61 subjects could not be included due to the exclusion criteria, and 47 individuals were excluded as they were not able to participate in pre-specified clinical follow-ups. Thus, 100 patients were included in the study, of which 33 men and 67 women [25]. No patient received lithium treatment and 26 subjects did not take any medications at all. Sertraline (*n* = 20), Citalopram (*n* = 12), Mirtazapine (*n* = 12), Venlafaxine (*n* = 9), and Fluoxetine (*n* = 7) were the most frequent medications prescribed [25]. Out of a total of 100 participants, DNA samples were extracted from 88 individuals. Four subjects later committed suicide (three by hanging, one by substance overdose)—information provided from matching unique identification numbers with the national Cause of Death register. Subjects were stratified into the severe suicidal phenotype if using a violent SA method (hanging, shooting, gassing or drowning) [12], exhibiting a Freeman scale score >6 [26], or if having later committed suicide.

### Blood sample collection, DNA methylation profiling and preprocessing

Blood samples were obtained by standard procedures. Subjects were not fasting at the time of extraction, which occurred in the morning. The phenol-chloroform method was implemented to retrieve DNA from 88 participants, and thereafter, subjected to bisulfite conversion according to the EZ DNA Methylation—GoldTM kit (ZymoResearch, USA). Resulting DNA was subsequently subjected to hybridization to the Illumina Infinium Methylation EPIC BeadChip. Array imaging by Illumina iScan system (Illumina, San Diego, CA, USA) followed, resulting in the quantification of methylation values across 850 K individual probes for all samples.

Methylation data was preprocessed in accordance with specialized instructions for EA estimation (https://dnamage.genetics.ucla.edu/) and associated recent publications [27]. The meffil package for R statistics (https://github.com/perishky/meffil/) was utilized for quality control (QC) and normalization of raw methylation IDAT data, making use of control probes to isolate biological from technical variation. Two samples failed QC and were excluded from the subsequent analysis (sample exclusions: 1 sample with incorrect sex prediction, 0 samples with sex detection outliers, 2 samples with an outlier in predicted median methylated vs unmethylated signal, 1 duplicate sample), resulting in 86 subjects for which methylation β-values were extracted.

### DNAm age calculation

27,253 CpG sites were uploaded to the DNAm Age Calculator (https://dnamage.genetics.ucla.edu/). This list contrasts with the recommended 30,085 long list of probes suggested by Horvath et al., in that 2,562 probes are unaccounted for. This inconsistency is explained by the non-complete CpG-site overlap between the EPIC and 450 K Illumina platforms—the present study pertaining to the EPIC chip and the construction of the epigenetic clocks being derived from the 450 K BeadChip [27]. To perform the ‘Advanced Analysis’, a complementary dataset was uploaded containing matched data on chronological age, gender and tissue type used to extract the DNAm data (whole blood).

Several EA measures were extracted for 86 individuals. These include the measures of intrinsic epigenetic age acceleration by Horvath and Hannum (IEAA [17] and IEAAHannum [18], respectively), extrinsic epigenetic age acceleration (EEAA [17], AgeAccelPheno [19] and AgeAccelGrim [20]—the two latter based on independent estimators [20, 28]). EA acceleration measures are directly interpreted in that a value greater than 0 implicates that EA is higher than chronological age, and vice-versa. Measures of intrinsic age acceleration (IEAA) were also extracted, unlike EAA representing an estimate independent from alterations in blood cell type proportions that have been related to aging [29]. The age-adjusted DNAmTL (DNAmTLadjAge) is based on telomere length, and is interpreted inversely in that a negative DNAmTLadjAge value implicates shorter than expected DNAm-based estimates of telomere length and implying accelerated EA. A detailed overview of the different epigenetic age predictors has been previously presented [30].

### Statistical analysis

The Mann–Whitney U-test, independent samples *t*-test or chi-squared test were utilized to detect between-group differences in demographic and clinical variables. Subsequently investigated measures of EA acceleration were analyzed for normality by Shapiro–Wilk test and were all normally distributed except for *AgeAccelerationResidualHannum*, the latter thus subjected to Blom-transformation [31] to achieve normality for univariate and multivariate analyzes (not used for visualization). To assess the accuracy of the epigenetic estimators used in this study, interrelatedness of epigenetic clocks and their association to chronological age was evaluated by Pearson correlations for the full cohort, and for each suicidal phenotype group separately [32, 33]. For illustrative purposes, the mean difference between GrimAge and chronological age was assessed for each group separately and the percentage change in relation to chronological age was visualized in violin plots. All demographic and clinical variables presented in (Table 1) were considered as co-variates in the subsequent multivariate analysis. To exclude bias from overfitting by including too many co-variates, we only included parameters strongly associated with AgeAccelGrim or exhibiting between-group differences relating to suicidal phenotype with a *p*-value < 0.05. Demographic and clinical characteristics were thus evaluated for an association with AgeAccelGrim by Pearson correlations for continuous variables and independent samples *t*-test for dichotomous variables. Substance dependence was significantly associated to AgeAccelGrim (*p* = 0.02788). No other demographic or clinical characteristics were significantly correlated with AgeAccelGrim. As such, gender (*p* = 0.005642) and occurrence of substance dependence (yes/no) were determined as co-variates for the subsequent multivariate model. Committed suicide exhibited significant between-group differences (*p* = 0.02375) but was not evaluated as a potential co-variate as this variable was already incorporated in the group stratification (see above). The power.t.test function for R statistics was implemented to evaluate power to detect meaningful differences in AgeAccelGrim in relation to suicidal phenotypes. Independent samples *t*-tests were implemented to investigate between-group differences in various EA measures by suicidal risk group. As violin plots were not indicative of any other direction of association, we implemented one-tailed hypothesis *t*-tests to evaluate whether EA acceleration measures were greater in the severe suicidal group. Hence, two-tailed hypotheses were not evaluated. Binomial logistic regression models were performed to estimate the probability of differentiating attempted suicide severity based on AgeAccelGrim and optimal co-variates, contrasting severity of suicide attempt to Grim Age Acceleration, gender, and occurrence of substance dependence (yes/no).

**Table 1:** Characteristics of subjects.

	Attempted suicide (*n* = 86)		
	High-risk group	Low-risk group	Statistics (*t*-test, Mann-Whitney U-test, Chisq. test), *p*-value)
*N*	30	56	
Age (years)	35.7 (12.1)	33.6 (12.3)	*ns*
Men: women, *n* (%)	16 (53.3): 14 (46.7)	12 (21.4): 44 (78.6)	**0.005642**
BMI, mean (SD)	24.5 (4.6)	24.9 (4.3)	*ns*
Depression, *n* (%)	23 (74.2)	37 (68.5)	*ns*
Borderline personality disorder, *n* (%)	7 (23.3)	5 (8.9)	*ns*
Other personality disorder, *n* (%)	8 (36.7)	9 (17.9)	0.09456
Alcohol dependence, *n* (%)	9 (26.7)	8 (16.1)	*Ns*
Substance dependence, *n* (%)	6 (19.4)	9 (15.8)	*Ns*
Completed suicide, *n* (%)	4 (13.3%)	0 (0.0)	**0.02375**
KIVS subscale, *n* (%)			
^1^Expressed violent behavior during			
Childhood	0 (0.0)	1 (1.8)	*ns*
Adulthood	6 (20.0)	4 (7.1)	*ns*
^2^Exposure to violent behavior during			
Childhood	10 (33.3)	15 (26.8)	*ns*
Adulthood	15 (50.0)	19 (33.9)	*ns*

Values are shown as mean (SD) unless otherwise specified. *p*-Values were calculated by means of *t*-test, Mann–Whitney *U*-test or chi-squared test, contrasting values for subjects in the high-risk vs low-risk suicide attempt group. A one-tailed *p*-value <0.05 was considered significant. *KIVS* Karolinska Interpersonal Violence Scale, *ns* not significant.

Significant findings in bold (*p* < 0.05).

## Results

### Baseline descriptives

Participants had a mean age of 34 years (SD = 12.2). Eighty-six percent of participants fulfilled criteria for one or more Axis I psychiatric diagnoses. A majority had a mood disorder (71%), whilst anxiety disorder (6%), adjustment disorder (5%) and alcohol dependence (4%) accounted for the rest of the psychiatric morbidity. 30% and 29% had comorbid personality disorder or substance-related disorders, respectively (alcohol dependence being the most common in the latter case). 30 subjects (34.9%) were stratified as severe suicide attempters. Male subjects were overrepresented in the high-risk group (*p* < 0.01). Other clinical characteristics were equally distributed across the suicidal groups, i.e., BMI, mood or anxiety disorders, prevalence of borderline or other personality disorder, alcohol dependence or substance abuse. There were no significant differences between the groups in scores on the Karolinska Interpersonal Violence Subscales (a measurement of exposure and expression of violent behavior during the lifespan). Significantly more participants in the high-risk group committed suicide at a later time point (*p* = 0.024) (Table 1).

### Interrelatedness between epigenetic clocks and their association to chronological age

**Fig. 1:**
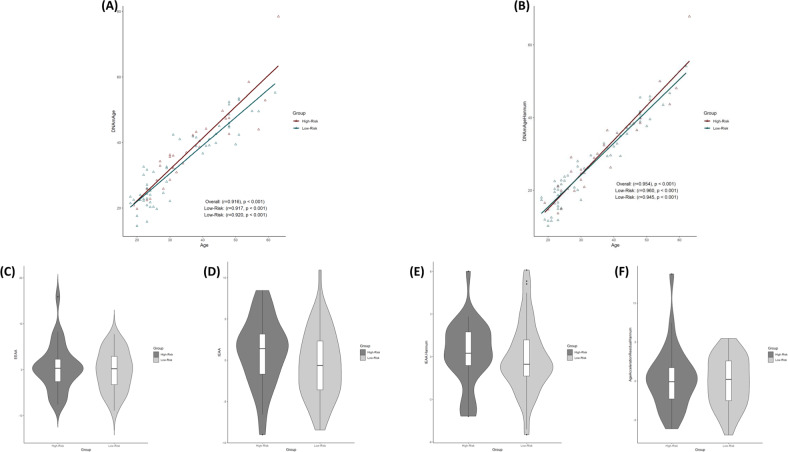
Horvath and Hannum epigenetic age acceleration. **A**, **B** Scatterplots show Horvath or Hannum Age vs chronological age. Pearson’s correlation analysis indicated a significant correlation between DNA methylation age and chronological age in both groups. **C–F** Violin plot with boxplots show Horvath EAA, IEAA, Hannum EAA, or EEAA. Between-group comparisons were conducted using a Student’s *t*-test. No significant between group differences were revealed (*p* > 0.1). *EAA* epigenetic age acceleration, *IEAA* intrinsic epigenetic age acceleration, *EEAA* extrinsic epigenetic age acceleration.

**Fig. 2:**
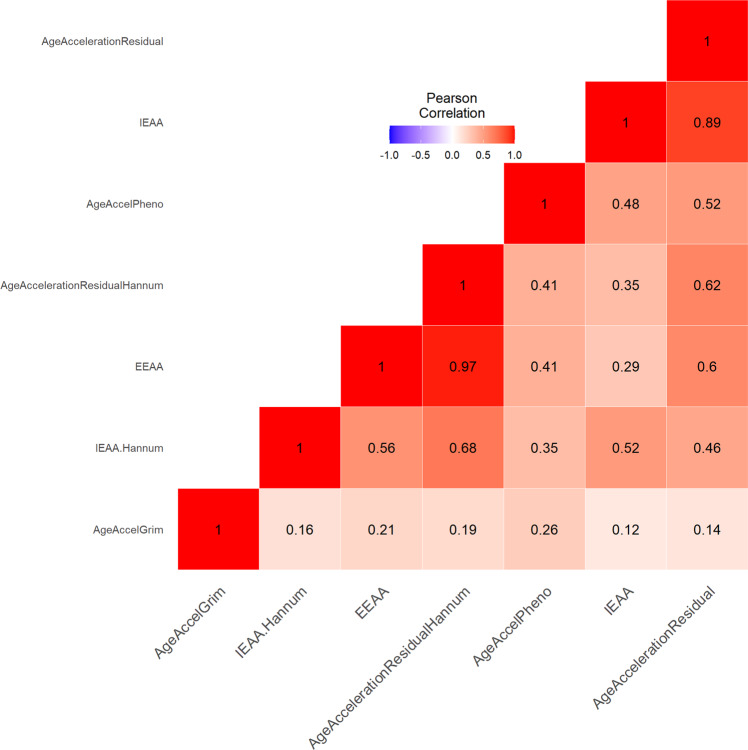
Correlations of AgeAccelGrim with other epigenetic age clocks. The plot visualizes the inter-correlations between seven DNAm clocks. The deeper color indicates stronger correlations. AgeAccelGrim was relatively independent to other clocks, while AgeAccelPheno was correlated of medium strength with the acceleration of Horvath and Hannum clocks.

**Fig. 3:**
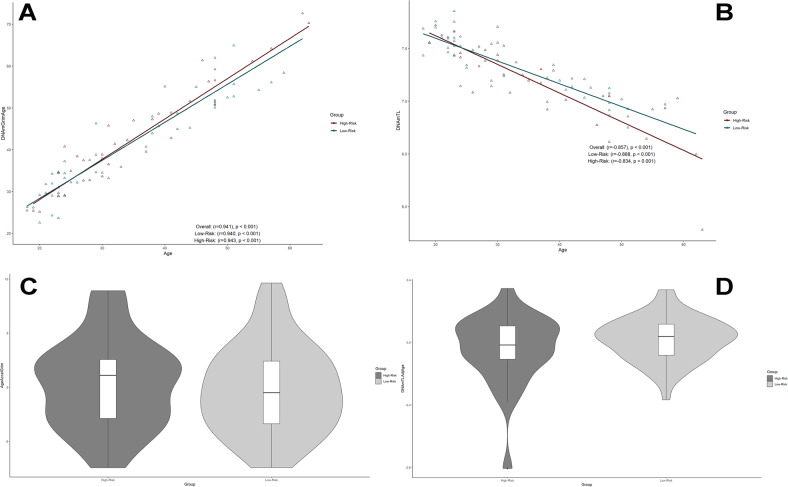
Grim epigenetic age acceleration and DNA methylation-based telomere length. **A**, **B** Scatterplots show GrimAge or DNAmTL vs. chronological age. Pearson’s correlation analysis indicated a significant correlation between GrimAge/DNAmTL and chronological age in both groups. **C**, **D** Violin plot with boxplots shows Grim EAA or DNAmTLAdjAge. Student’s *t*-test showed no significant between-group differences. EAA, epigenetic age acceleration; DNAmTL, DNA methylation-based telomere length; DNAmTL AdjAge, age-adjusted DNAmTL.

There was a significant correlation between chronological age and Horvath Age (*r* = 0.916, *p* < 0.001), Hannum Age (*r* = 0.954, *p* < 0.001), GrimAge (*r* = 0.941, *p* < 0.001), and DNAmTL (*r* = −0.857, *p* < 0.001) (Figs. 1 and 3). The strong linear relationships between each DNAm age/DNAmTL and chronological age indicated a valid high accuracy of the epigenetic estimator used in this study. AgeAccelGrim exhibited a small but significant strength of correlation with AgeAccelPheno (r = 0.26, *p* = 0.017), which resembles previous findings [20, 22]. AgeAccelGrim was relatively independent to other clocks, while AgeAccelPheno was correlated of medium strength with the acceleration of Horvath and Hannum clocks. Horvath and Hannum clocks were strongly correlated amongst themselves (r > 0.5, *p* < 0.001), including both intrinsic and extrinsic measures of epigenetic age acceleration (Fig. 2).

### Relatedness of *AgeAccelGrim* and other epigenetic age acceleration clocks with severity of suicide attempt

The baseline mean difference between GrimAge and chronological age for the full cohort averaged 7.3 years (SD = 4.14 years). Mean EA increases were similarly greater in the low- (mean difference = 7.1 years, SD = 4.19 years) and high-risk group (mean difference = 7.5 years, SD = 4.15 years). The percentage change in baseline DNAmGrimAge compared to chronological age in this cohort of suicide attempters averaged 23.4% and 25.2% in the low- and high-risk group, respectively (Supplementary Fig. 1). Power-analysis shows that the study was sufficiently powered to detect differences of 2.2 in AgeAccelGrim between suicidal risk-groups for a desired power of 0.8 in one-tailed hypothesis *t*-tests (Supplementary Fig. 2.), and 2.5 for two-tailed hypothesis *t*-tests (which was not evaluated). In our primary analysis, we assessed associations of epigenetic age acceleration to severity of suicide attempt by univariate analyses using various estimators of biological age. These analyses uniformly implicated that epigenetic age acceleration is independent of severity of suicide attempt, i.e., EAA, IEAA, Hannum IEAA, AgeAccelerationResidualHannum, AgeAccelGrim and DNAmTLadjAge were not associated with severity of suicide attempt by independent sample’s *t*-tests (*p* > 0.1) (Figs. 1 and 3). A binomial logistic regression model contrasting severity of suicide attempt to Grim Age Acceleration with optimal co-variates did not reveal any association related to AgeAccelGrim (*p* > 0.1) (Table 2).

**Table 2:** Binomial logistic regressions contrasting severity of suicide attempt to Grim Age Acceleration.

	Estimate	Std. Error	Z-value	*P*-value
Intercept	0.31694	0.4236	0.748	0.45433
AgeAccelGrim	0.01023	0.06293	0.163	0.87088
Gender (Female)	−1.44223	0.50452	−2.859	**0.00426**
Substance dependence	−0.13404	0.66066	−0.203	0.83922

Binomial logistic regression contrasting severity of suicide attempt to Grim Age Acceleration, Gender and Substance Dependence. Shown are the coefficients and *p*-values. Significant findings in bold (*p* < 0.05).

## Discussion

This is the first study to investigate epigenetic age acceleration (EAA) in relation to severity of suicide attempt in a well-characterized cohort of suicide attempters. EAA is hypothesized to be a plausible surrogate variable to assess whether the high risk/severe phenotype exhibits increased morbidity and mortality conferred by cellular senescence. In this cohort of suicide attempters, DNAmGrimAge exceeded chronological age by 7.3 years on average for the full cohort, conferring a mean 24.6% increase of EA in relation to actual age. Implementing the state-of-the-art EA algorithms [20] in a study sufficiently powered to detect differences exceeding 2.2 years, we demonstrate that severity of suicide attempt is not related to epigenetic age acceleration markers that have previously been demonstrated to strongly predict physiological dysregulation and mortality risk. These findings implicate that the excess in all-cause mortality bestowed by suicide attempts is largely unaffected by suicide attempt method (violent or non-violent), thus, also unrelated to putative neurobiological underpinnings that have been implicated in severe suicidal behavior [9–11]. Taken together, the present study demonstrates that suicide attempters exhibit a biological strain as measured by EA, making them more susceptible to prematurely contracting severe physical illnesses (i.e., medical conditions strongly predicted by DNAmGrimAge: time-to-CHD, time-to-cancer (any), and time-to-other age-related medical conditions)—the major contributing factor to all-cause mortality in suicide attempters [6]. The observed independency of EAA in relation to severity of suicide attempt—a plausible surrogate variable for predicting biological strain and non-suicidal mortality—implicate that the cumulative increase in non-suicidal excess-mortality observed after suicide attempts is conferred by exogenous factors that are unaffected by severity of attempt.

Excess morbidity and all-cause mortality conferred by mental disorders and suicide attempts are significant and underrecognized major health concerns [1]. Alarmingly, suicide attempters have been previously demonstrated to exhibit substantial reductions in life expectancies (11–20 years), largely due to somatic co-morbidities and other factors unrelated to death by suicide [6]. Harmful lifestyle practices have been ascribed as major contributors to these adverse health outcomes [2, 3]. Considering recent studies that implicate severe suicidal behavior to exhibit unique pathophysiological mechanisms in comparison to the less severe phenotype, morbidity- and mortality-preventive measures could be improved by understanding 1) whether individuals with such features exhibit intrinsically accelerated biological aging conferred by inherited factors, or 2) whether disadvantageous lifestyle practices secondary to psychiatric disease and/or suicide attempts have a more substantial influence. The global association of AgeAccelGrim to measures of substance dependency supports the notion that disadvantageous lifestyle practices and/or shared unfavorable genetic factors confer most of the excess all-cause mortality observed in suicide attempters, irrespective of suicide attempt method (violent or non-violent). Thus, recognizing a history of suicide attempt (irrespective of method or severity inherent in the act itself) as a risk-factor for overall somatic ill-health [20], could help facilitate somatic screening and early recognition, implementation of primary preventative efforts and adequate secondary treatment (when somatic disease is manifest) [34, 35], which holds potential to improve prognosis of comorbid conditions [36, 37] and, ultimately, reduce the substantial excess in non-suicidal mortality in these populations. Notably, primary prevention of asymptomatic subjects of CHD and cancer (any)—both DNAmGrimAge-associated causes of mortality—involves the promotion of a healthy lifestyle throughout life emphasizing physical activity, diet, tobacco cessation and, to some extent, sunscreen use [34, 35]. Importantly, the present findings indicate that practitioners should be acting equally powerfully to prevent and treat somatic comorbidities in all suicide attempters, irrespective of severity inherent in the act itself.

This study is burdened by several limitations. First, the cross-sectional design prevents any assured inferences of causality. A longitudinal study-design would have allowed for analyzing the dynamic quality of EA in relation to severity of suicidal behavior. Second, as the study material was devoid of non-suicidal psychiatric controls, decisively assessing epigenetic age acceleration conferred by any suicide attempt was not feasible. Studies including matched non-suicidal psychiatric controls are needed to fully elucidate the putative contribution of suicide attempts to EA. Third, power-calculations implicated that the study was not sufficiently powered to exclude small cumulative increases/decreases in excess mortality conferred by more severe suicide attempts. However, the present study would be sufficiently powered to detect clinically significant EA differences. Assessing EA disparities of small magnitude would require a sizeable cohort of suicide attempters and would, arguably, be unlikely to be relevant to inform clinical practice. Fourth, while the relatively limited sample size prevented adjustments for medication use, the only medications prescribed to more than five individuals were antidepressants. Thus, it can be argued that the study is well-suited to curtail any major influence from unmeasured sources of confound from medication use, as there were no between-group differences in any psychiatric disorder necessitating the prescription of such medications. Moreover, while the global DNA methylation pattern pertains to over 850 000 CpG-sites of which some have been associated with antidepressant drug use [38], the epigenetic age estimators used in the present study are based on only a small fraction of these (i.e. 27,253 CpG-sites, https://dnamage.genetics.ucla.edu/). Using a multivariable model investigating associations of DNAmGrimAge acceleration to antidepressant drug use in 1099 African American subjects, Kho et al. could not evince any effect from antidepressant drug use on GrimAge-derived EA measurements [39]. Thus, albeit it cannot be assuredly excluded, it should be highly unlikely that the findings presented in the present manuscript are biased by confound from medication use. Fifth, a separate analysis for subjects that subsequently committed suicide would have been of interest. However, the reliable interpretation of any such post-hoc analysis is prevented by the relatively small number of individuals (*n* = 4). Study strengths include the representative patient population of suicide attempters with thorough diagnostics of the psychiatric disorders and a careful assessment of severity of suicidal behavior as well as the consideration of possible confounders such as gender, childhood adversity, and comorbidity patterns. Previous studies found no differences concerning prevalence of smoking and substance use between violent and non-violent suicide attempters at age 18 [10]. Moreover, the statistical methods used allow for using peripheral tissues to reliably estimate EA—epigenetic age estimators have been previously demonstrated to be accurately measured from almost any tissue of the body [40].

In conclusion, our results show that epigenetic age acceleration markers previously demonstrated to strongly predict physiological dysregulation and mortality are not related to severity of suicide attempt. These findings suggest that suicidal behavior associated excesses in morbidity and all-cause mortality is largely conferred by harmful lifestyle patterns and/or shared unfavorable genetic factors [41]. Taken together, these findings indicate that a history of suicide attempt (irrespective of method or severity inherent in the act itself) could be considered as a risk-factor for prematurely contracting severe physical illnesses (i.e., CHD, cancer (any)). Such endeavors cold facilitate early recognition, implementation of primary preventative efforts and adequate treatment, when manifest [34, 35], which holds potential to improve prognosis of comorbid conditions [36, 37] and, ultimately, reduce the substantial excess in non-suicidal mortality in these populations.

## Supplementary information

Supplementary Figure titles and legends

Supplementary Figure 1

Supplementary Figure 2

## Data Availability

The data underlying the findings presented in this study are available upon reasonable request.
