# Supplementary material for: Accelerated epigenetic aging in suicide attempters uninfluenced by high intent-to-die and choice of lethal methods
Source: Transl Psychiatry. 2022 Jun 2;12:224. doi: 10.1038/s41398-022-01998-8 (PMC9163048; doi:10.1038/s41398-022-01998-8)
Supplement: Supplementary file 1 — Supplementary Figure titles and legends [file 41398_2022_1998_MOESM1_ESM.docx]

**Supplementary Figures: Titles and legends**

**Supplementary Figure 1. Difference between baseline *DNAmGrimAge* and chronological age, expressed as percentage of chronological age**

**Figure legend**: Violin plot with boxplots show differences between *DNAmGrimAge* and chronological age, expressed as a percentage of chronological age. The percentage increase in epigenetic age compared to chronological age in this cohort of suicide attempters averaged 23.4% and 25.2% in the low- and high-risk group, respectively.

**Supplementary Figure 2. Sample size requirements for a power of 0.8 as a function of differences in *DNAmGrimAge***

**Figure legend**: The power.t.test function for R statistics was implemented to evaluate power to detect meaningful differences in AgeAccelGrim between suicidal phenotypes. As violin plots were not indicative of any other direction of association, we specified one-tailed hypothesis *t*-tests for the power calculations (evaluating whether EA acceleration measures are greater in the severe suicidal group). Power-analysis show that the study was sufficiently powered to detect differences of 2.2 in *AgeAccelGrim* between suicidal risk-groups for a desired power of 0.8 in one-tailed hypothesis *t*-tests (**Supplementary Figure 2.**), and 2.5 for two-tailed hypothesis *t*-tests (data not illustrated).
