# Supplementary figures and images for: Accelerated epigenetic aging in suicide attempters uninfluenced by high intent-to-die and choice of lethal methods
Source: Transl Psychiatry. 2022 Jun 2;12:224. doi: 10.1038/s41398-022-01998-8 (PMC9163048; doi:10.1038/s41398-022-01998-8)

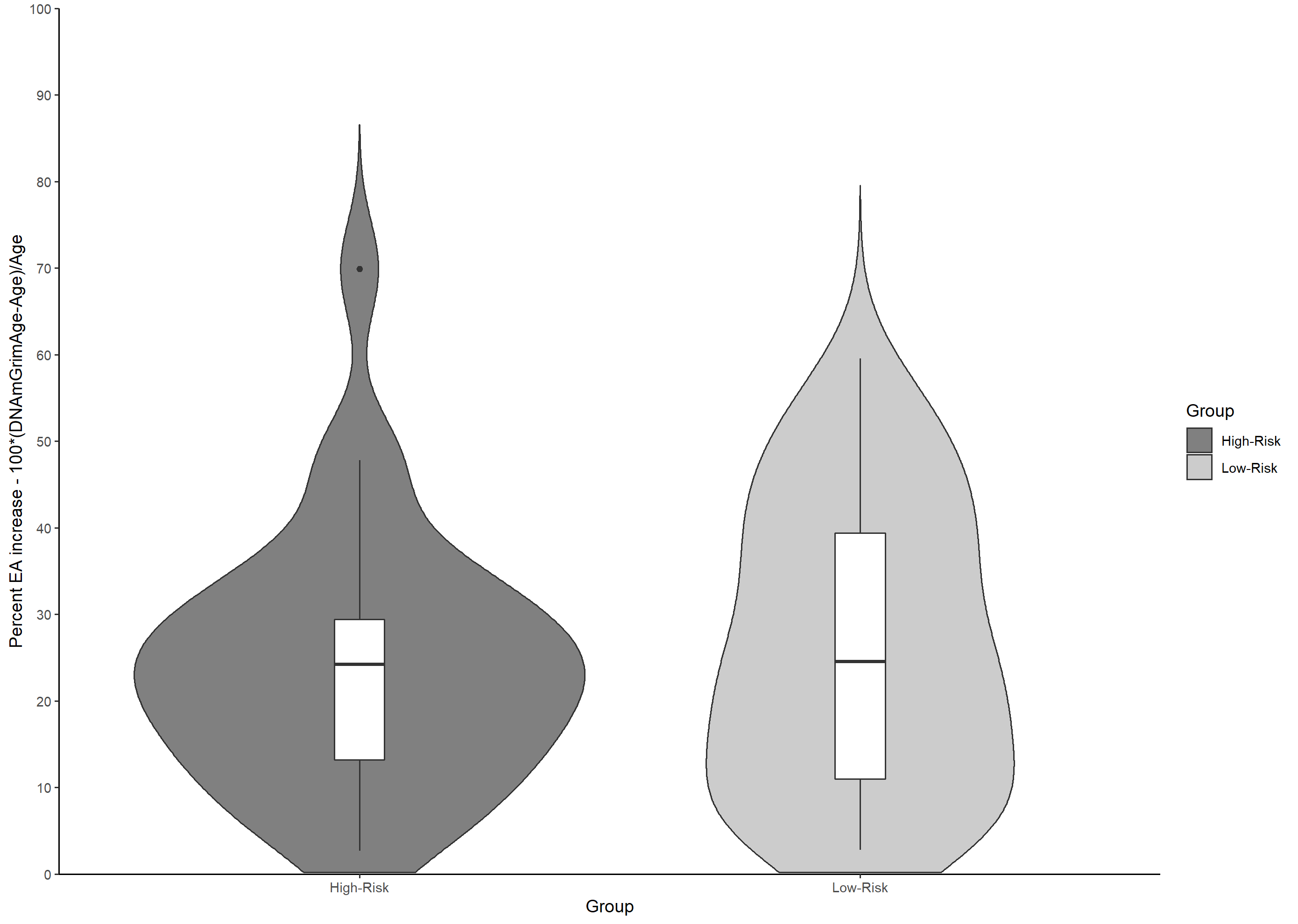

Supplement: Supplementary file 2 — Supplementary Figure 1 [file 41398_2022_1998_MOESM2_ESM.png]

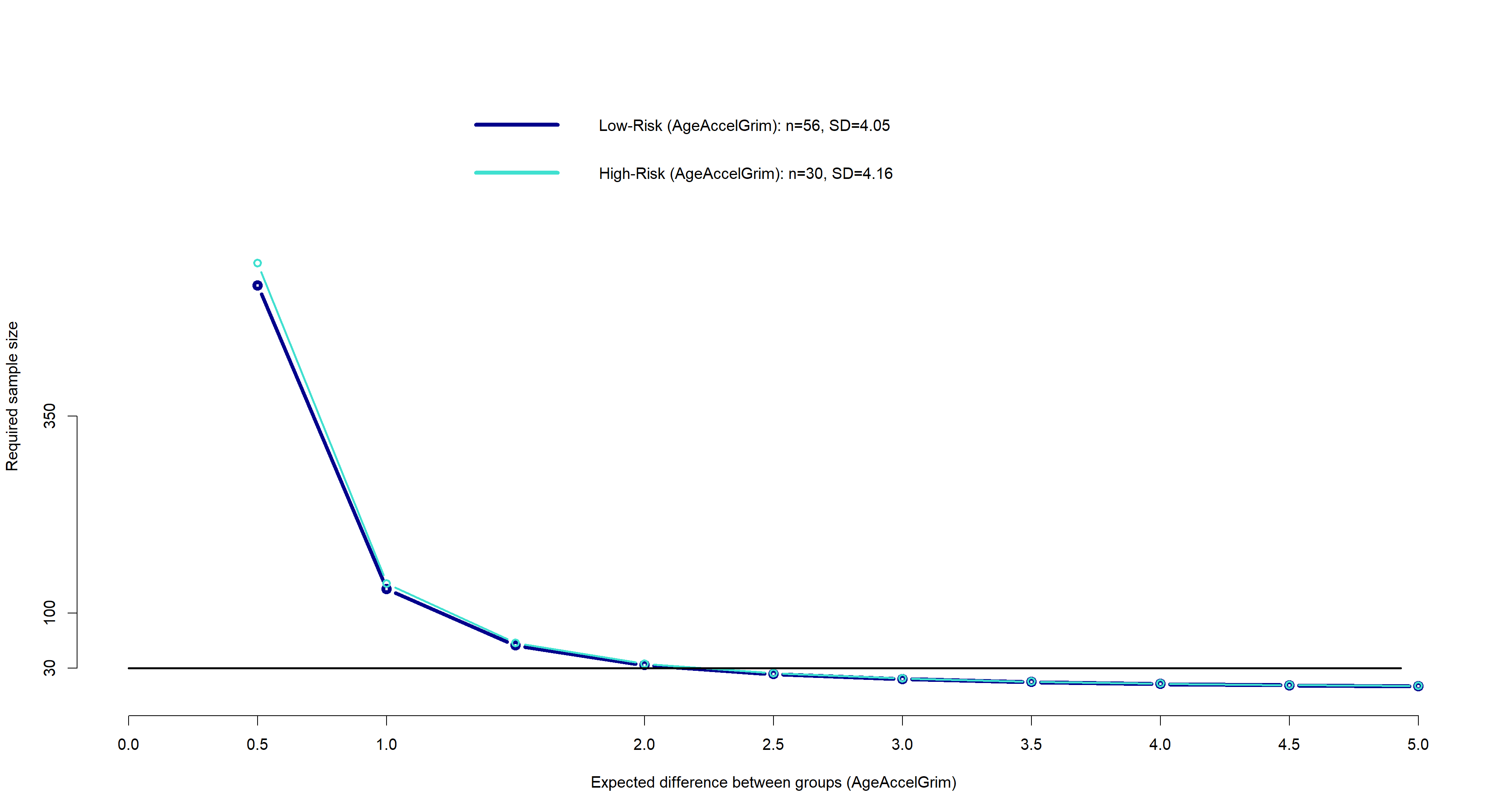

Supplement: Supplementary file 3 — Supplementary Figure 2 [file 41398_2022_1998_MOESM3_ESM.png]
